# A new method for the calculation of functional and path integrals

**DOI:** 10.1038/s41598-023-40750-0

**Published:** 2023-08-24

**Authors:** Amos A. Hari, Sefi Givli

**Affiliations:** Faculty of Mechanical Engineering, Technion - Israel Institute of Technology, Haifa, Israel

**Keywords:** Mechanical engineering, Statistical physics

## Abstract

This paper addresses a disconnect between the pivotal role of functional (path) integrals in modern theories, such as quantum mechanics and statistical thermodynamics, and the currently limited ability to perform the actual calculation. We present a new method for calculating functional integrals, based on a finite-element formulation, which solves all limitations of existing methods. This approach is far more robust, versatile, and powerful than the prevailing methods, thus allowing for more sophisticated computations and the study of problems that could not previously be tackled. Importantly, existing procedures, element libraries and shape functions, which have been developed throughout the years in the context of engineering analysis and partial differential equations, may be directly employed for this purpose.

## Introduction

Functional (or path) integrals, are ubiquitous in a wide range of physical and mathematical problems, ranging from quantum mechanics to statistical thermodynamics through biology, chemistry, engineering and finance^1–9^. Perhaps the most famous functional integral is related to the notion of path integral in quantum mechanics, introduced by Richard Feynman^1^, which generalizes the action principle of classical mechanics. Functional integrals generalize the notion of integration over *vector spaces* to integration over *function spaces*. Much like vector integration, $\int f({{\textbf {v}}}){\textrm{d}}{{{\textbf {v}}}}$, where the integral is the sum of all volume elements in the integration space each weighted by an integrand *function*, functional integration, $\int g[u]{\mathscr{D}}u$, is the summation process over all admissible functions each weighted by an integrand *functional*. Currently, the prevailing method for evaluating path-integrals is a “slicing method” which involves a naive discretization of the spatial (or temporal) space followed by summation over the function values at the discrete points^1–3^. The use of this approach requires, for example, that the derivatives of the state function with respect to the independent coordinates are approximated using finite-differences. Based on this “slicing method” and similar ones, path integrals have been calculated for a wealth of systems and scenarios that appear in quantum mechanics, statistical thermodynamic, Coulomb systems, polymer physics, particle orbits, quantum statistics, superfluids, financial markets, and more (see, for example, the the comprehensive textbook by Kleinert^10^ and the references therein). These methods are straight forward and useful, however they are limited to 1-D systems in time or space, or to very simple and regular spatial geometries, such as rectangles. Further, even in 1-D, the application of certain boundary conditions, as well as constraints inside the domain, can be cumbersome. Thus, there is a clear need in a new, more sophisticated, method for the calculation of functional integrals.

Although functional integrals have been mostly applied to 1-D, line-like, objects^10^ (embedded in a 2-D or 3-D space), they have also been calculated for 2-D and 3-D objects. However, in all these cases simplifying assumptions were adopted in order to reduce the calculation to that of a 1-D object or by assuming a very simple and regular geometry that enables the use of “standard” methods such as the slicing method mentioned above. For example, the theoretical study of the behavior of fluid membranes has often avoided the calculation of 2-D functional integrals by considering simplistic 1-D models or by restricting the analysis to the most probable scenario associated with “classic” energy minimizers^11–14^. In other studies, where the membrane was considered a 2-D object subjected to random fluctuations, free energy was evaluated by means of 2-D functional integrals; however, the geometry of the membrane was assumed to be extremely simple in order to enable the calculation. For example, the stochastic behavior of lipid membranes was studied by considering a simple rectangular “patch” subjected to periodic boundary conditions, which in turn allows for expressing the free energy in Fourier space^15,16^, or by assuming the geometry of a perfect sphere to express the functional integral in terms of spherical harmonics^17,18^. Rectangular patches were also studied using a slicing-like method by means of, e.g., a simple rectangular-grid discretization^19^, two-level rectangular grid discretization for studying the interaction of inclusions on a lipid membrane^20^, or an equilateral triangle discretization^21,22^ adopted for 2-D square membranes. In a somewhat different context, the free energy of 3-D elastomeric networks also requires the calculation of path integrals. This, however, involves a 1-D path integral for a single chain (a line-like object embedded in a 3-D space) followed by volume averaging for the entire network^23^.

We propose a new approach for the calculation of functional integrals that is based on the finite-element (FE) formulation rather than the aforementioned naïve discretization. The FE method is a numerical approximation method predominantly used for solving partial differential equations (PDEs) in all fields of engineering, ranging from stress analysis and heat transfer in structures to electromagnetic scattering of objects and the design of photonic crystals^24–28^. Driven by the growing need to tackle more complicated engineering problems, the FE method has become the standard numerical tool for engineering design and mechanical analysis, thanks to its generality, robustness and versatility. Still, the use of the FE approach for evaluating functional integrals has been largely overlooked, practically limiting the computation of path integrals to simple geometries. Interestingly, the idea of using finite elements to compute path integrals and partition functions has been mentioned in the context of filament networks^29^, although in a limited form. There, the entropic elasticity of the network was studied by considering it as a truss-like structure that is comprised from line-like “elements”. Thus, partition function, free energy, and thermo-mechanical properties of the network were expressed in terms of a stiffness matrix. In this context, the current paper generalizes the notion of using finite-elements for calculating functional integrals of objects comprised from “discrete” line-like members to continuum objects of any geometry and physics. The progression of these ideas resembles the evolution of finite elements in engineering, from a method to analyze simple truss and frame structures to one that applies for continuum problems in the broad field of mechanical design, engineering, and beyond.

As we show below, applying the FE approach to functional integrals requires some technical care; however, the powerful formulation opens the door for more sophisticated computations and for the study of problems that could not previously be tackled. In that sense, it may be reminiscent of the revolution brought by the FE approach to partial differential equations, which has enabled solving complex engineering problems with complicated geometries and all types of boundary conditions or constraints. Further, although the method we propose is new, the underlying foundations are mature and well-established: Countless papers and textbooks have been published on the theory of FE^24,25,30–33^, and highly sophisticated computational schemes and software have been developed; all may readily be repurposed for our needs. For example, one may directly use the large established libraries of finite elements and associated shape-functions^34^, or apply well-established meshing procedures and related software^35^. Perhaps the most important advantage that the FE formulation introduces to the computation of functional integrals is the preservation of the state functions as functional entities; this is unlike the existing approaches where the state functions are reduced to a finite set of discrete points. This property of the method allows for a more rigorous treatment and valuable insights. Moreover, even in simple cases, where the formulation does not provide fundamentally new results, we may still find ourselves appreciating the refreshing interpretations the method gives, or by the words of Richard P. Feynman *there is a pleasure in recognizing old things from a new point of view*^2^.

## Results

### The method

Consider a stochastic system whose (micro)state is described by the real valued function $u(x)\in V$, where *V* is the space of all possible states, or admissible functions, of the system, and *x* is a point in a parameter space $\Omega$. At the moment don’t make any assumption on the class of continuity of *V*. Let the functional *p*[*u*] , with $p:V\rightarrow {\mathbb {R}}$, be the probability density corresponding to state *u*(*x*) ; and let *g*[*u*] be some functional, $g:V\rightarrow {\mathbb {R}}$, dependent on the system’s state. The average of the state-dependent functional *g*[*u*] is defined as the sum of *g*[*u*] over all possible states of the system weighed by the respective probability $p[u]{\mathscr {D}}u$, hence expressed as a *functional integral*

1 $$\begin{aligned} \left\langle g[u] \right\rangle = \int _V g[u]p[u]{\mathscr{D}}u. \end{aligned}$$

The mathematical rigor for such operation is quite subtle, but the concept is well established, e.g., the Feynman path integral^1–3^; in that context, Eq. (1) should be regarded as a formal way to express the functional averaging process.

**Figure 1:**
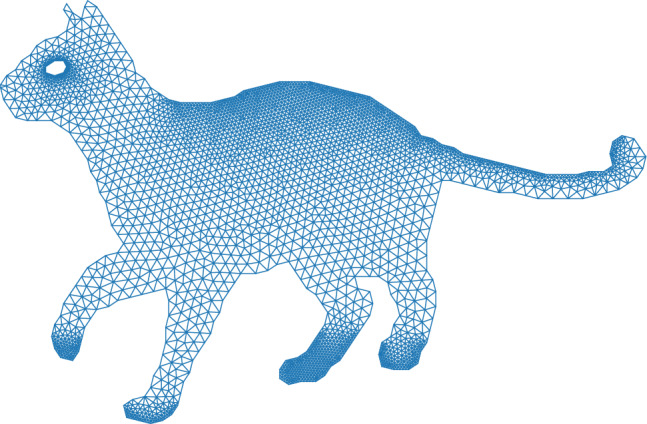
Example of a non-uniform mesh over a 2-D domain in the shape of a cat’s silhouette. The mesh was generated using the open-source Gmsh^35^.

We hereby present a new method for the calculation of functional integrals, based on a FE formulation. As a first step, we approximate the function space *V* by introducing a subspace $V^h\subset V$. The superscript *h* is called the *mesh parameter* and it implies that the functions $u^h\in V^h$ are associated with a *mesh*, or a discretization, of the domain $\Omega$ (see for example Fig. 1 for a cat-geometry domain). The mesh parameter, *h*, is a measure for the size of the largest element in the mesh, therefore when $h\rightarrow 0$ then $V^h\rightarrow V$. Following standard FE practice, we define $u^h\in V^h$ as follows

2 $$\begin{aligned} u(x) \approx u^h(x) = \sum _{A\in \eta } \phi _A(x) d_A + \sum _{A\in \eta _u}\phi _A(x){\bar{u}}_A. \end{aligned}$$

Here $\eta$ is the set of *open nodes*, i.e. nodes where *u* is variable, and $\eta _u$ is the set of *closed nodes*, i.e. nodes where *u* is prescribed. The functions $\phi _A(x)$ are called FE shape functions; these are continuous functions that are related to the mesh, in the sense that each function $\phi _A(x)$ gets the value of one at node *A* and vanishes in all elements that don’t contain that node. It is therefore apparent that the coefficients $d_A$ and ${\bar{u}}_A$ are the values of *u* at the respective node. It is shown in FE theory that as $h\rightarrow 0$ then $u^h\rightarrow u$ and the approximation error in (2) is given by $\Vert u-u^h\Vert = Ch^\alpha$, where $\alpha$ is the rate of convergence which depends on the type of FE shape functions used^24^.

Once we are able to represent *u* by a finite number of degrees of freedom (DOFs), we may substitute the approximation $u^h$ into the probability density and obtain $p^h({{\textbf {d}}})$. Note that $p^h$ is a *function* of the variables ${{\textbf {d}}}=\left\{ d_A\right\}$, rather than a *functional*, and it is normalized such that $\int p^h({{\textbf {d}}}){\textrm{d}}{{{\textbf {d}}}}=1$. Similarly, we express $g^h({{\textbf {d}}}) = g[u^h]$ as a function of ${{\textbf {d}}}$. Since ${{\textbf {d}}}$ uniquely determines $u^h$ and $g^h$, then the problem of finding $\left\langle g[u^h] \right\rangle$ reduces to computing $\left\langle g^h({{\textbf {d}}}) \right\rangle$, where

3 $$\begin{aligned} \left\langle g^h({{\textbf {d}}}) \right\rangle = \int _{{\mathbb {R}}^{N}} p^h({{\textbf {d}}}) g^h({{\textbf {d}}}){\textrm{d}}{{{\textbf {d}}}}. \end{aligned}$$

Above, *N* is the number of DOFs (the cardinal number of $\eta$) and ${\mathbb {R}}^{N}$ is the space of all real vectors of length *N*. Equation (3) is thus the FE approximation of the functional integral (1), and the finite-dimensional integration can be carried out analytically (where possible) or numerically. The Markov-Chain Monte-Carlo method is especially appropriate for this task, because its rate of convergence is independent on *N* and it is particularly suitable for finding the statistical moments of complicated distributions^36^.

Note that the method described here can be readily generalized for vector functions ${{\textbf {u}}}={{\textbf {u}}}(x)$ by writing Eq. (2) separately for each component of ${{\textbf {u}}}$. Further, the domain $\Omega$ may be a simple 1-D interval, as in the case of the path integral in quantum mechanics where the coordinate *x* above represents time, or be a multi-dimensional domain of any geometry, such as in the case of statistical thermodynamics of a 3-D body. Unfortunately, the “slicing method”, commonly adopted for computing path integrals, cannot be applied to the latter. In terms of the proposed method, however, the only difference between these two cases is the use of different finite elements and corresponding shape functions; while in the 1-D case the elements are lines (or 1-D segments) and the shape functions are described in terms of one coordinate, in 2-D the elements have a 2-D geometry, such as triangles or quadrilaterals, and the corresponding shape functions are described using two coordinates. Similarly, if the domain $\Omega$ is three-dimensional, 3-D elements are used, etc. It is noted that the use of a non-uniform mesh, where the domain $\Omega$ is divided into elements of different sizes, is a standard practice of the FE method as illustrated in Fig. 1. This allows, for example, to use a finer mesh in regions where high accuracy is needed. This feature is another important attribute of the versatile and powerful finite-element formulation. Finally, the physics of the problem dictates the number of DOFs at each node of the element. This is exemplified in the two examples below, where the first example involves one DOF at each node, while in the second example two DOFs are used at each node.

### Example-I: a string at finite temperature

Consider a string of length *L* and uniform tension $\sigma$ with both ends held fixed at a horizontal level. A lateral force *f*(*x*) is distributed along the string, and the entire system is submerged in a heat reservoir of temperature *T*. Let $u(x)\in V$ describe the transverse displacement of the string at $x\in \Omega =[0,L]$ and regard *u* as the state of the system. We would like to find, for example, the average state of the system. The space $V=\left\{ u|u\in H^{1}(\Omega ), u(0)=u(L)=0\right\}$ is the set of all square-integrable functions over $\Omega$ with square-integrable first derivatives (Sobolev space) that admit the boundary conditions $u(0)=u(L)={\bar{u}}=0$. The probability density corresponding to micro-state *u* is^37^

4 $$\begin{aligned} p[u] = \frac{1}{Z}e^{-\beta E[u]}, \end{aligned}$$

where the partition function, *Z*, is a normalization constant, $\beta =(k_BT)^{-1}$, and *E*[*u*] is the energy functional5with $u_{,x}=\partial u/\partial x$. Accordingly, the average state of the system is given by the functional integral

6 $$\begin{aligned} \left\langle u \right\rangle = \frac{1}{Z}\int _V u e^{-\beta E[u]} {\mathscr{D}}u. \end{aligned}$$

Following the method described above, we write the finite-element approximation as

7 $$\begin{aligned} u\approx u^h = \sum _{A\in \eta } \phi _Ad_A + \sum _{A\in \eta _u} \phi _A{\bar{u}}_A, \end{aligned}$$

where for simplicity, $\phi _A(x)$ are linear FE shape functions^24^, and thus *u* is approximated by $u^h$ in a continuous piece-wise-linear manner (For a detailed derivation, of this step and the steps that follow, the reader is referred to the worked-example file in the Supplementary Material). Next, we substitute the FE approximation $u^h$ into the energy functional *E*[*u*] so it becomes a function $E^h({{\textbf {d}}})=E[u^h]$ of the variable ${{\textbf {d}}}$. This energy integral is calculated at the *element level*. Thus, define the vectors ${\varvec{\upphi }}(x)=\left\{ \phi _1^e(x), \phi _2^e(x)\right\} ^T$ of the element shape functions and ${{\textbf {d}}}^e=\left\{ d_1^e, d_2^e\right\} ^T$ of the element state values, such that the state in each element is given by $u^h = {\varvec{\upphi }}^T{{\textbf {d}}}^e$. Then, rewrite the integral (5) as a sum of integrals over $\Omega ^e\subset \Omega$, the domain of the *e*-th element. The energy of that element is then

8 $$\begin{aligned} (E^h)^e = \frac{1}{2}{{\textbf {d}}}^{eT}{{\textbf {k}}}^e{{\textbf {d}}}^e - \hat{{{\textbf {f}}}}^{eT}{{\textbf {m}}}^e{{\textbf {d}}}^e, \end{aligned}$$

where $\hat{{{\textbf {f}}}}^e=\left\{ {\hat{f}}^e_1,{\hat{f}}^e_2\right\} ^T$ is a vector whose components are the values of *f* at the element nodes, such that $f\approx f^h={\varvec{\upphi }}^T\hat{{{\textbf {f}}}}^e$ describes the force inside the element. The symmetric matrices ${{\textbf {k}}}^e=\sigma \int _{\Omega ^e}{\varvec{\upphi }}_{,x}{\varvec{\upphi }}_{,x}^T{\textrm{d}}{\Omega }$ and ${{\textbf {m}}}^e=\int _{\Omega ^e}{\varvec{\upphi }}{\varvec{\upphi }}^T{\textrm{d}}{\Omega }$ are respectively called the element stiffness and mass matrices and they are calculated in each element separately; however, for many elements, including the 1-D element considered here, a formula for these matrices is readily found in the literature^24,34^. Next, define the matrix ${{\textbf {K}}}$, vector ${{\textbf {v}}}$ and scalar *S* such that the expression for the global energy becomes

9 $$\begin{aligned} E^h({{\textbf {d}}})=\frac{1}{2}{{\textbf {d}}}^T{{\textbf {K}}}{{\textbf {d}}}+({{\textbf {v}}}-{{\textbf {F}}})^T{{\textbf {d}}} + \frac{1}{2}S. \end{aligned}$$

Here ${{\textbf {K}}}$ is the *global* stiffness matrix, the vectors ${{\textbf {v}}}$ and ${{\textbf {F}}}$ are related to the contribution of prescribed displacements and of external loads, respectively, and the scalar *S* corresponds solely to the contribution of boundary condition to the energy. This quadratic energy may now be substituted into Eq. (4) so it becomes an off-centered Gaussian and thus $\left\langle {{\textbf {d}}} \right\rangle$ may be calculated analytically from Eq. (3) by considering the particular case of $g^h({{\textbf {d}}})={{\textbf {d}}}$.

An interesting particular case of the above example was studied by Van Lear and Uhlenbeck^38^, where the variance of the random deflection, attributed to thermal undulations, was obtained for $f=0$. There, rather than considering functional integrals, the authors formulated and solved the equations of motion that include a fluctuating term (force), and found that

10 $$\begin{aligned} \textrm{Var}(u(x)) = \frac{L}{\beta \sigma }\left( \frac{x}{L}-\frac{x^2}{L^2}\right) . \end{aligned}$$

**Figure 2:**
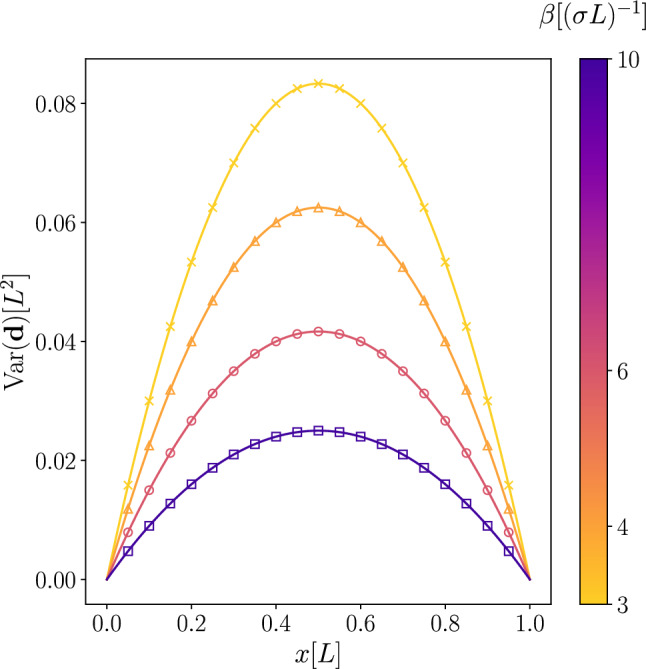
The variance of the deflection of a string plotted for different temperatures. The analytical result (solid lines) obtained by Van Lear and Uhlenbeck^38^ is compared with the numeric results (markers) obtained using the proposed method.

In our example, taking $f=0$ and ${\bar{u}}=0$ simplifies the approximate energy function into $E^h({{\textbf {d}}}) = \frac{1}{2}{{\textbf {d}}}^T{{\textbf {K}}}{{\textbf {d}}}$. Therefore, the mean state is $\left\langle {{\textbf {d}}} \right\rangle ={{\textbf {0}}}$, the covariance is $\textrm{Cov}({{\textbf {d}}}) = \left\langle {{\textbf {d}}}{{\textbf {d}}}^T \right\rangle = \beta ^{-1}{{\textbf {K}}}^{-1}$, and thus the variance of the deflection at the nodes is $\beta ^{-1}\textrm{diag}({{\textbf {K}}}^{-1})$. Results of this analysis, compared to the solution obtained by Van Lear and Uhlenbeck, are presented in Fig. 2 and Table 1.

**Table 1:** The numerical values of the calculated and the analytical results shown in Fig. 2 for $\beta =10[(\sigma L)^{-1}]$

Node number	Node position $[L]$	Calculated $\times 10^{-3} [L^2]$	Exact $\times 10^{-3} [L^2]$	Absolute error $[L^2]$
1	0.05	4.7500	4.75	$3.47\times 10^{-18}$
2	0.10	9.0000	9.00	$1.04\times 10^{-17}$
3	0.15	12.7500	12.75	$2.43\times 10^{-17}$
4	0.20	16.0000	16.00	$3.82\times 10^{-17}$
5	0.25	18.7500	18.75	$5.90\times 10^{-17}$
6	0.30	21.0000	21.00	$6.94\times 10^{-17}$
7	0.35	22.7500	22.75	$6.25\times 10^{-17}$
8	0.40	24.0000	24.00	$5.90\times 10^{-17}$
9	0.45	24.7500	24.75	$5.90\times 10^{-17}$
10	0.50	25.0000	25.00	$5.20\times 10^{-17}$
11	0.55	24.7500	24.75	$4.16\times 10^{-17}$
12	0.60	24.0000	24.00	$2.08\times 10^{-17}$
13	0.65	22.7500	22.75	$1.04\times 10^{-17}$
14	0.70	21.0000	21.00	$1.04\times 10^{-17}$
15	0.75	18.7500	18.75	$6.94\times 10^{-18}$
16	0.80	16.0000	16.00	$3.47\times 10^{-18}$
17	0.85	12.7500	12.75	$3.47\times 10^{-18}$
18	0.90	9.0000	9.00	$1.73\times 10^{-18}$
19	0.95	4.7500	4.75	$6.07\times 10^{-18}$

### Example-I.A: a string at finite temperature with non-quadratic energy

To give better idea regarding the use of the method when the energy is not quadratic, we consider once more the system of Example-I, however for this instance we do not take the quadratic approximation for the energy of a string, and instead consider the full energy expression11If the rotations are small, i.e. $|u_{,x}| \ll 1$, then the square-root in the tension term may be approximated using its first-order Taylor expansion, and Eq. (11) would simplify back into (5), up to an additive constant.

We advance using the energy from equation (11) and the same tactic presented in Example-I. First, the state is approximated using finite elements, $u\approx u^h$; then, the energy integral is decomposed and calculated over each element domain $\Omega ^e$ separately yielding the following element-energy

12 $$\begin{aligned} (E^h)^e = \int _{\Omega ^e} \sigma \sqrt{1+({\varvec{\upphi }}_{,x}^T{{\textbf {d}}}^e)({\varvec{\upphi }}_{,x}^T{{\textbf {d}}}^e)}{\textrm{d}}{\Omega } - \hat{{{\textbf {f}}}}^{eT}{{\textbf {m}}}^e{{\textbf {d}}}^e. \end{aligned}$$

If linear shape functions are considered, as in Example-I, then using the fact that the derivatives for the linear shape function are constant, we get that

13 $$\begin{aligned} E^h({{\textbf {d}}}) = \sum _{e=1}^{N_{el}} \left[ \sigma |\Omega ^e|\sqrt{1 + (\sigma |\Omega ^e|)^{-1}{{\textbf {d}}}^{eT}{{\textbf {k}}}^e{{\textbf {d}}}^e} - \hat{{{\textbf {f}}}}^{eT}{{\textbf {m}}}^e{{\textbf {d}}}^e\right] , \end{aligned}$$

where $N_{el}$ is the number of elements in the mesh and $|\Omega ^e|$ is the length of the *e*-th element. The element matrices ${{\textbf {k}}}^e$ and ${{\textbf {m}}}^e$ in Eqs. (12) and (13) are the same as those defined for Eq. (8) in Example-I. In cases where the shape functions are not linear, simplifying the element integral as was done in Eq. (12) is not necessarily possible, and the element integral should be calculated using a deterministic quadrature rule, such as Gauss-Legendre quadrature. Plugging the energy expression (13) into Eq. (3), would result in an *N*-D integral that has to be calculated numerically. As mentioned in the discussion following Eq. (3), the Monte-Carlo method is a natural candidate for this purpose.

### Example-I.B: a membrane at finite temperature

Consider the following expansion of Example-I to 2-D. Instead of a string, we consider a membrane of tension $\sigma$ that is subjected to a lateral force $f({{\textbf {x}}})$ and the entire system is submerged in a heat reservoir of temperature *T*. Let $u({{\textbf {x}}})\in V$ describe the transverse displacement of the string at ${{\textbf {x}}}\in \Omega$ and regard *u* as the state of the system. The shape of the unloaded membrane, $\Omega$, could be completely general, e.g., the domain depicted in Fig. 1. The space $V = \left\{ u|u\in H^1(\Omega ), u|_{\partial \Omega }=0\right\}$ is the 2-D version of the function space defined for Example-I, however now the domain of the functions, $\Omega$, is two-dimensional, and their boundary value is given over the boundary of the domain, $\partial \Omega$. The energy of the membrane is given by14The only other detail that changes for the 2-D system with respect to its 1-D counterpart, is that we need to take two-dimensional shape functions in order to represent the FE approximation. For specificity we choose triangular elements, although other 2-D elements may be chosen. Accordingly, we define the vectors ${\varvec{\upphi }}({{\textbf {x}}})=\left\{ \phi _1^e({{\textbf {x}}}),\phi _2^e({{\textbf {x}}}),\phi _3^e({{\textbf {x}}})\right\} ^T$ of the element shape functions and ${{\textbf {d}}}^e = \left\{ d_1^e,d_2^e,d_3^e\right\} ^T$ of the element state values. Following the same steps as in Example-I, the element stiffness matrix is redefined as ${{\textbf {k}}}^e=\sigma \int _{\Omega ^e}({\varvec{\upphi }}_{,x}{\varvec{\upphi }}_{,x}^T+{\varvec{\upphi }}_{,y}{\varvec{\upphi }}_{,y}^T){\textrm{d}}{\Omega }$, but other then that, the definition for ${{\textbf {m}}}^e$, and the expressions for the element and global energies, are symbolically identical to those obtained in Example-I. This demonstrates the power of the method, and that a generic 2-D domain can be handled with the exact same general mechanism that was developed for 1-D. The extension of this to 3-D is trivial.

### Example-II: a beam at finite temperature

Consider a stochastic system that is modeled by an Euler–Bernoulli beam of length *L* and uniform bending stiffness $K_B$ with one end fixed and the other free. A lateral load *f*(*x*) is distributed along the beam, and the entire system is submerged in a heat reservoir of temperature *T*. Let $u(x)\in V$ describe the transverse deflection of the beam at $x\in \Omega =[0,L]$ and regard *u* as the state of the system. The space $V=\left\{ u|u\in H^{2}(\Omega ), u(0)=0, u_x(0)=0\right\}$ is the set of all square-integrable functions over $\Omega$ with square-integrable first and second derivatives that admit the fixed boundary conditions $u(0)=0,\ u_x(0)=0$. Similar to the previous example, we want to find the average state of the system. The statistical distribution is given by Eq. (4), with the energy functional15The fundamental difference compared to the previous example is that for the energy (15) to be well defined, we demand stronger requirements on the class of continuity of *u*, namely that $V\subset H^2$. Accordingly, $u^h\in V^h$ must satisfy these continuity requirements. To this end, we approximate *u* using the Hermite cubic shape functions $\phi _{(Ai)}$ where $i=1,2$^24,34^. These shape functions and their derivative vanish everywhere, except in the elements that share node *A*. Moreover, at node *A*, the shape functions satisfy $\phi _{(A1)}=\phi _{(A2),x}=1$ and $\phi _{(A2)}=\phi _{(A1),x}=0$. This property allows dictating separately the displacements (*u*) and rotations $(u_{,x})$ at the element nodes, thus

16 $$\begin{aligned} u\approx u^h = \sum _{i=1}^{N_{\textrm{ndof}}}\left( \sum _{A\in \eta ^{(i)}} \phi _{(Ai)}d_{(Ai)} + \sum _{A\in \eta _u^{(i)}}\phi _{(Ai)}{\bar{u}}_{(Ai)}\right) . \end{aligned}$$

Here $N_{\textrm{ndof}}$ is the number of nodal DOFs (in our case, $N_{\textrm{ndof}}=2$). The values of *u* at the nodes are $d_{(A1)}$ and the values of $u_{,x}$ at the nodes are $d_{(A2)}$, the sets $\eta ^{(i)}$ and $\eta _u^{(i)}$ are defined as in Eq. (2) but they include only nodes with open or closed *i*-th DOF. Define the vector ${\varvec{\upphi }}(x)=\left\{ \phi _{(11)}^e(x), \phi _{(12)}^e(x),\phi _{(21)}^e(x), \phi _{(22)}^e(x)\right\} ^T$ of the element shape functions and the vector ${{\textbf {d}}}^e=\left\{ d_{(11)}^e, d_{(12)}^e,d_{(21)}^e, d_{(22)}^e\right\} ^T$ of the element state values, such that the state in each element is given by the cubic function $u^h = {\varvec{\upphi }}^T{{\textbf {d}}}^e$. The energy expression for the element is the same as in Eq. (8), other than ${{\textbf {k}}}^e = K_B\int _{\Omega ^e}{\varvec{\upphi }}_{,xx}{\varvec{\upphi }}_{,xx}^T{\textrm{d}}{\Omega }$; therefore the global energy also has the quadratic form of Eq. (9) and $\left\langle {{\textbf {d}}} \right\rangle$ may be calculated analytically from Eq. (3).

### Numerical example: adhesion of elastic body to a rigid substrate

In what follows, we present numerical results obtained using the proposed finite-element formulation. The model considered is prototypical to phenomena such as detachment of biological cells, peeling of a thin film from a substrate, etc., and demonstrates how the formulation can be conveniently applied to complex systems composed of coupled linear and non-linear elements. We emphasize that while the model may be suitable for describing real phenomena, such as those mentioned above, it is presented here merely for demonstrating the proposed method; thus justification of the model and its assumptions are not further discussed.

**Figure 3:**
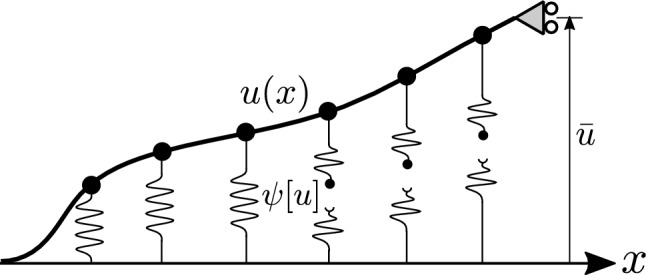
An illustration of the system under consideration. An Euler-Bernoulli beam is fixed on its left, supported on its right and connected throughout to a rigid substrate via breakable bonds. The deflection of the beam is described by the function *u*(*x*) , and the potential of the bonds by the functional $\psi [u]$ in Eq. (17). A displacement ${\bar{u}}$ is prescribed on the simply-supported end, and the response of the system at finite temperature is investigated.

**Figure 4:**
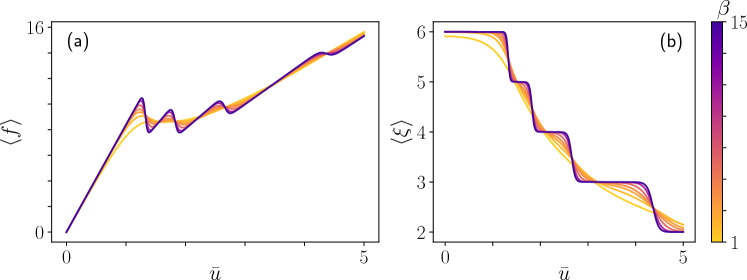
The response of the system at different temperatures. (**a**) Relation between the end-displacement ${\bar{u}}$ and corresponding mean force $\left\langle f \right\rangle$ for various temperatures. As the temperature rises (lower $\beta$), the decohesion process becomes smoother due to the rise in the probability of previously improbable states. (**b**) The mean number of attached bonds $\left\langle \xi \right\rangle$ as function of the prescribed displacement ${\bar{u}}$. As in the force-displacement case, the detachment process becomes smoother with the rise of the temperature.

Consider an Euler–Bernoulli beam of the sort described in the previous example, but instead of a lateral distributed force, the non-fixed end of the beam is supported at a height ${\bar{u}}$. Thus,

$$\begin{aligned} V = \left\{ u \Big | u\in H^{2}(\Omega ), u(0)=0, u_x(0)=0, u(L)={\bar{u}}\right\} . \end{aligned}$$

The beam is adhered to a rigid substrate as illustrated in Fig. 3. The adhesion is modeled by a set of *N* bonds connected at points $x_A\in \Omega$ ($A=1,\ldots ,N)$ along the beam. When the *A*-th bond is connected it acts as a linear spring of stiffness $k_A$, and when it is broken it exerts no force. Hence we use the following potential function to describe the adhesion

17 $$\begin{aligned} \psi _A[u] = {\left\{ \begin{array}{ll} \frac{1}{2}k_A(u(x_A))^2 &{}{\rm connected}\\ \frac{1}{2}k_AU_A^2 &{}{\rm broken} \end{array}\right. }. \end{aligned}$$

Here $U_A$ is constant of units length that describes the broken state potential in terms of elongation of the spring. Note that due to the stochastic nature of our system, each bond may break and reconnect randomly. Accordingly, the state of each bond, either connected or broken, is identified by a two-state *spin variable*. A similar, yet simpler, adhesion-decohesion model was introduced and discussed by Florio et al.^39^. There, it was suggested to introduce a single *N*-state spin variable, $\xi$, for the entire array of bonds. This is based on the assumption that due to the one-sided decohesion process we have $\xi$ connected bond at the fixed-end-side and $N-\xi$ broken bonds at the supported-end-side. The potential energy of the system is therefore

18 $$\begin{aligned} E[u;\xi ] = \int _{\Omega } \frac{1}{2}K_B u_{xx}^2{\textrm{d}}{\Omega } + \sum _{A=0}^{\xi }\frac{1}{2}k(u(x_A))^2 + \frac{1}{2}(N-\xi )kU^2 \end{aligned}$$

The next step is to approximate *u* using Hermite cubic shape functions as was done in the previous example. In principle, one may use any mesh as long as it has nodes at all the $\left\{ x_A\right\}$ points where the beam is attached to the substrate through a breakable bond. For simplicity, we consider here a mesh with *N* unknowns located where the springs are connected. The approximate energy function is then

19 $$\begin{aligned} E^h({{\textbf {d}}};\xi ) = \frac{1}{2}{{\textbf {d}}}^T\left( {{\textbf {K}}}+{{\textbf {K}}}_{\mathrm {conc.}}(\xi )\right) {{\textbf {d}}} + {{\textbf {v}}}^T{{\textbf {d}}} + \frac{1}{2}\left( S + (N-\xi )\eta \right) \end{aligned}$$

where ${{\textbf {K}}}$, ${{\textbf {v}}}$ and *S* are defined as usual, and ${{\textbf {K}}}_{\mathrm {conc.}}$ is a matrix full of zeros except for $kU^2$ at $\xi$ entries along the diagonal corresponding to connected bonds. The statistical distribution of the system is $p[u;\xi ] = \exp (-\beta E[u;\xi ])/Z$, and the discretized version is the well-studied Gaussian distribution. Once we have $p^h({{\textbf {d}}},\xi )$ we essentially *know everything* about the system, and we can calculate some interesting statistical properties and study how they are influenced by temperature. For example, Fig. 4a shows the effect of temperature on the force-displacement relation of the mean force, $\left\langle f \right\rangle = -(\partial \log (Z^h)/\partial {\bar{u}})/\beta$, applied by the support with respect to the prescribed displacement ${\bar{u}}$. We may also calculate $\left\langle \xi \right\rangle$, the mean number of connected bonds, as a function of ${\bar{u}}$, as shown in Fig. 4b. These results are given in *non-dimensional* form after the energy was rescaled by $E_0=K_BU^2/L^3$ and lengths were rescaled by *U*; the calculations were carried out with the values: $kU^2/E_0 = 5$, $\beta = \left\{ 15,10,6,4,3,2,1\right\} E_0$ and $N=6$. For reference, and future use of interested readers, the Python code that generates these results is provided in the Supplementary Material.

A few comments are in order. Note that, using the proposed method, we can naturally and easily choose to fix or load any DOFs. To this end, one only needs to modify the identity of the relevant DOFs in the data array. On the other hand, using existing methods, the addition of constraints inside the domain requires tremendous technical effort which involves manually modifying the matrices that describe the discrete system. For example, if the beam is also rigidly supported at the midspan, the existing methods require either splitting the domain and adding some continuity conditions, or completely reformulate the problem to include the new constraint. On the other hand, the proposed method takes care of the additional constraint automatically since each element is treated separately and then all assembled to a global matrix, as was described in Eqs. (8) and (9). Also, using the proposed method, the analysis can be directly extended to 2-D, thus considering the adhesion of a surface, of any geometry, rather than a 1-D beam. This is similar to what was done in Example-I.B above. Technically, it requires meshing a 2-D domain (see Fig. 1) and updating the data arrays that identify the elements along with the procedures that calculate their local stiffness matrix. Other than that, the algorithm remains identical, leading to the same mathematical structure as in the 1-D case. Such analysis, of a 2-D surface with arbitrary geometry, is impossible with existing methods.

## Discussion

We presented a new method for calculating functional integrals based on finite-elements formulation. The new approach complements existing methods in the sense that these methods may be more convenient/useful for deriving closed-form analytical expression for objects with regular geometry. On the other hand, the proposed method is more suitable for complicated problems and non-regular geometries since it is far more robust, versatile and powerful than any prevailing methods; it allows the calculation of functional integrals over any domain subjected to any boundary conditions or constraints, while not limited to 1-D objects like the “slicing method” or to very simple and regular 2-D/3-D objects, such as a simple rectangle or a sphere. Due to the nature of the discretization, a finer mesh may be used in regions where high accuracy is needed. Moreover, by employing the FE formulation, the functional identity of the state-function is naturally maintained throughout the calculation, enabling insightful perspectives even in 1-D. Just as importantly, existing finite-element routines, elements libraries and shape functions, which have been developed throughout the years for solving PDEs, can be directly employed for calculating functional integrals as well. Three illustrative examples have been discussed, demonstrating the formulation for single and multiple nodal DOFs and showing that the formulation can be conveniently applied to complex systems, even with non-linear behavior. All in all, it is evident that the powerful FE formulation, which revolutionized the numerical analysis of PDEs, combined with modern computing power opens a door for new research opportunities by enabling the study of new problems which could have not been addressed before. Finally, as a secondary effect, the method is likely to accelerate the development and incorporation of new functional-integration schemes as independent modules in existing open-source and commercial FE software.

### Supplementary Information

Supplementary Information.

## Data Availability

All data generated or analysed during this study are included in this published article and its supplementary information files. The supplementary information files also include the code by which the data was generated.
