# Supplementary material for: A new method for the calculation of functional and path integrals
Source: Sci Rep. 2023 Aug 24;13:13852. doi: 10.1038/s41598-023-40750-0 (PMC10449871; doi:10.1038/s41598-023-40750-0)
Supplement: Supplementary file 1 — Supplementary Information. [file 41598_2023_40750_MOESM1_ESM.zip › numeric_example.pdf]

numeric_example

March 19, 2023

[1]:
```
%load_ext autoreload
%autoreload 2
```

[2]:
```
import numpy as np
import matplotlib
import matplotlib.pyplot as plt
import cmasher
import functions as u
from functions import MyPlot
```

[3]:
```
##########################################################
#                                                        #
#      Functional Integration using finite elements      #
#                                                        #
##########################################################

#########################################
# Domain defenition and mesh generation
#########################################
Nen = 2 # number of element nodes
Nel = 7 # number of elements
Nnp = Nel+1 # number of nodal points
Nsd = 1 # number of space dimension
Ndf = 2 # number of nodal DOFs

L = 1 # lenght of domain

nodes = np.linspace(0, L, Nnp)[:,None] # array of nodes, size=(Nnp, Nsd)
IEN = np.array([[i, i+1] for i in range(Nel)],
               dtype=np.int32).T # element connectivity array, Identity of␣
 ↪Element Nodes, 0-based indexing
ibc = np.zeros((Ndf,Nnp), dtype=np.int32) # identit of boundary condition, 0␣
 ↪for open, 1 for closed
ibc[0,0] = 1; ibc[1,0] = 1; ibc[0,-1] = 1

ID = np.zeros((Ndf,Nnp), dtype=np.int32) # Identity array; in: node number and␣
 ↪DOF, out: variable number
```

```
                                                 # outputs -1 if the DOF is closed.
aID = np.zeros((Ndf,Nnp), dtype=np.int32) # anti-Identity array; in: node␣
 ↪number and DOF, out: closed DOF number
                                                  # outputs -1 if the DOF is open.

A = 0
B = 0
for Ahat in range(Nnp):
    for i in range(Ndf):
        if (ibc[i,Ahat] == 0):
            ID[i,Ahat] = A
            aID[i,Ahat] = -1
            A += 1
        else:
            ID[i,Ahat] = -1
            aID[i,Ahat] = B
            B += 1
N = ID.max()+1 # N is the number of variables
Ncn = aID.max()+1 # number of closed nodes

LM = np.zeros((Ndf, Nen, Nel), dtype=np.int32) # location matrix
LM[:,:,:] = ID[:,IEN]
aLM = np.zeros((Ndf, Nen, Nel), dtype=np.int32) # anti-location matrix
aLM[:,:,:] = aID[:,IEN]
```

0.0.1 Pretty force-disp curves

[4]:
```
N = ID.max()+1 # N is the number of variables
Xi = Nnp-2 # number of springs - rough discretization - all open nodes have a␣
 ↪spring
beta = np.array([1.5e1, 1e1, 6e0, 4e0, 3e0, 2e0, 1e0])[::-1]
eta = 5
yd = np.linspace(0,5,250)
ubar = np.zeros((Ndf,Nnp)) # Helmhotlz condition
F = np.zeros(N) # force vector

f = np.zeros((yd.shape[0], 1+Ncn, beta.shape[0]))
xi = np.zeros((yd.shape[0], 2, beta.shape[0]))

for j in range(beta.shape[0]):
    print(f"j = {j}")
    for i in range(yd.shape[0]):
        ubar[0,-1] = yd[i]

        sol, XI, fmean, XImean = u.FE_an(Ncn, N, Xi, Nnp, Nel, Nen, Ndf,
                                         nodes, IEN, eta, beta[j], ubar, F, ID,␣
 ↪LM, aID, aLM)
```

```
        xi[i,:,j] = yd[i], XImean
        f[i,:,j] = yd[i], *fmean
```

```
j = 0
j = 1
j = 2
j = 3
j = 4
j = 5
j = 6
```

[5]:

```
with MyPlot(rcParams={'figure.dpi':100, 'text.usetex':True}) as pp:
    norm = matplotlib.colors.Normalize(
        vmin=np.min(beta),vmax=np.max(beta)) # define norm class
    cmap = cmasher.get_sub_cmap('plasma_r', 0.1, 0.9) # choose color map
    sm = matplotlib.cm.ScalarMappable(cmap=cmap, norm=norm) # create a␣
 ↪ScalarMappable

    for j in range(beta.shape[0]):
        plt.plot(f[:,0,j], f[:,-1,j], linestyle='-', color=sm.to_rgba(beta[j]))

    plt.xlabel(r"$\bar{u}$", fontsize=20)
    plt.ylabel(r"$\left<f\right>$", fontsize=20)

    plt.xticks(np.arange(0,6), labels=['$0$', *['' for i in range(4)],'$5$'],␣
 ↪fontsize=18)
    plt.yticks(np.arange(0,17,2), labels=['$0$', *['' for i in␣
 ↪range(7)],'$16$'], fontsize=18)

    clb = plt.colorbar(sm)
    clb.ax.set_title(r'$\beta$', fontsize=20, horizontalalignment='left')
    clb.ax.set_yticks(np.arange(1,16,2), labels=['$1$', *['' for i in␣
 ↪range(6)],'$15$'], fontsize=18)
    plt.tight_layout()
```

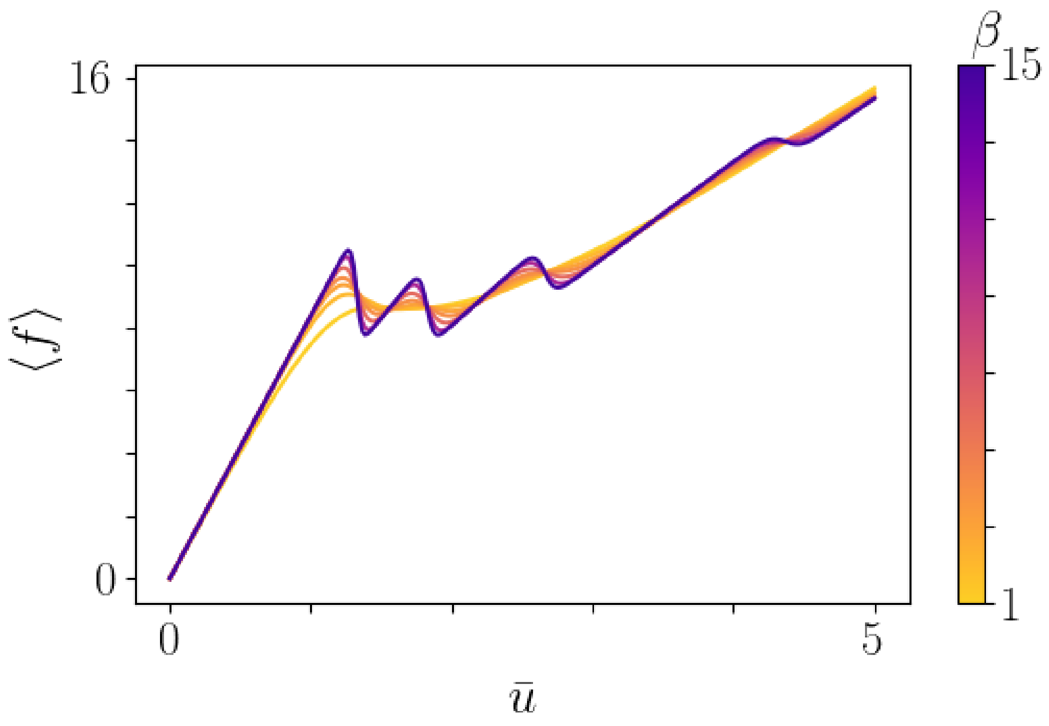


```
[6]: with MyPlot(rcParams={'figure.dpi':100, 'text.usetex':True}) as pp:
        norm = matplotlib.colors.Normalize(
            vmin=np.min(beta),vmax=np.max(beta)) # define norm class
        cmap = cmasher.get_sub_cmap('plasma_r', 0.1, 0.9) # choose color map
        sm = matplotlib.cm.ScalarMappable(cmap=cmap, norm=norm) # create a␣
     ↪ScalarMappable

        for j in range(beta.shape[0]):
            plt.plot(xi[:,0,j], xi[:,-1,j], linestyle='-', color=sm.
     ↪to_rgba(beta[j]))

        plt.xlabel(r"$\bar{u}$", fontsize=20)
        plt.ylabel(r"$\left<\xi\right>$", fontsize=20)

        plt.xticks([0,1,2,3,4,5], ['$0$','','','','','$5$'], fontsize=18)
        plt.yticks([2,3,4,5,6], fontsize=18)

        clb = plt.colorbar(sm)
        clb.ax.set_title(r'$\beta$', fontsize=20, horizontalalignment='left')
        clb.ax.set_yticks(np.arange(1,16,2), labels=['$1$', *['' for i in␣
     ↪range(6)],'$15$'], fontsize=18)
        plt.tight_layout()
```

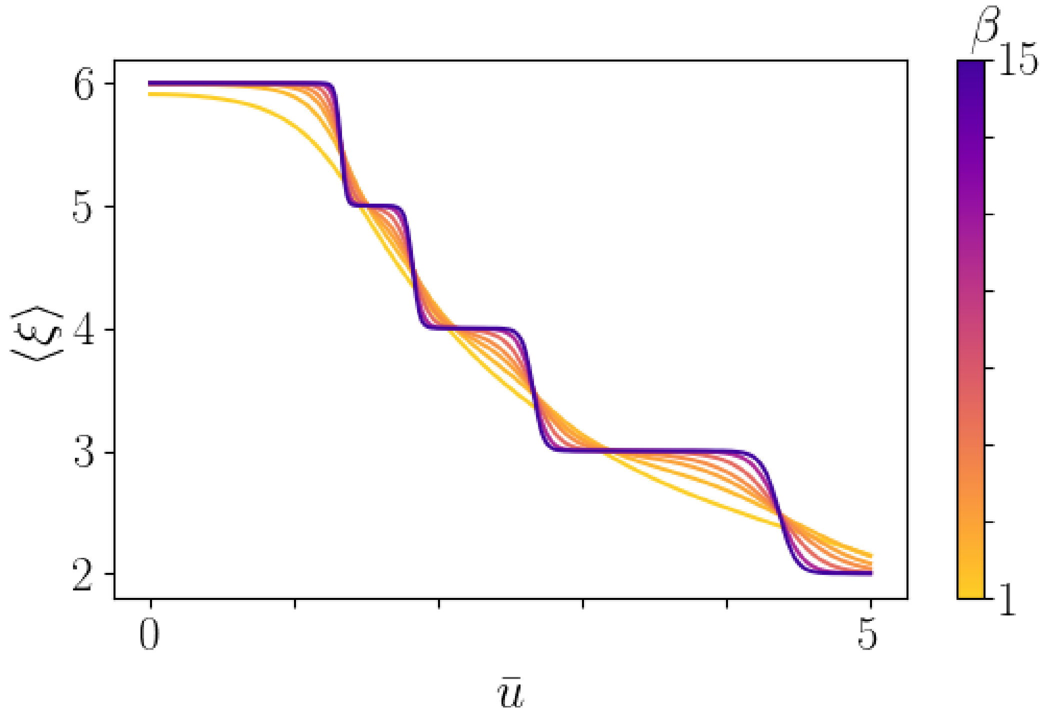

$\langle\xi\rangle$
6
5
4
3
2
0
5
$\bar{u}$
β
15
1
