# Supplementary material for: A new method for the calculation of functional and path integrals
Source: Sci Rep. 2023 Aug 24;13:13852. doi: 10.1038/s41598-023-40750-0 (PMC10449871; doi:10.1038/s41598-023-40750-0)
Supplement: Supplementary file 1 — Supplementary Information. [file 41598_2023_40750_MOESM1_ESM.zip › WorkedExample_Appendix.pdf]

A New Method for the Calculation of Functional and Path Integrals

Amos A. Hari[1] and Sefi Givli[1,*]

[1]Faculty of Mechanical Engineering, Technion - Israel Institute of Technology
[*]Corresponding Author: givli@technion.ac.il

Supplementary material: worked example

This SM (Supplementary material file) includes a complete and detailed development for the simple example of a fluctuating string from the main paper. The purpose of this appendix is to help readers that are unfamiliar with the finite element (FE) method make the transition from equation (7) to (8) to (9) in the main paper, and thus introduce them to the basic concepts of FEs. Readers who wish to fruther delve into the details of the FE method, are referred to the many existing books and online tutorials on the subject[1, 2, 3, 4, 5].

Consider a string of length L and uniform tension σ with both ends held fixed at a horizontal level. A lateral force $f(x)$ is distributed along the string, and the entire system is submerged in a heat reservoir of temperature T. Let $u(x) \in V$ describe the transverse displacement of the string at $x \in \Omega = [0, L]$ and regard u as the state of the system. We would like to find, for example, the average state of the system. The space $V = \{u | u \in H^1(\Omega), u(0) = u(L) = 0\}$ is the set of all square-integrable functions over Ω with square-integrable first derivatives (Sobolev space) that admit the boundary conditions $u(0) = u(L) = \bar{u} = 0$. The probability density corresponding to micro-state u is [6]

$$p[u] = \frac{1}{Z} e^{-\beta E[u]}, \tag{1}$$

where the partition function, Z, is a normalization constant, $\beta = (k_B T)^{-1}$, and $E[u]$ is the energy functional

$$E[u] = \underbrace{\int_\Omega \frac{1}{2} \sigma u_{,x}^2 \, d\Omega}_{\text{tension}} - \underbrace{\int_\Omega f u \, d\Omega}_{\text{load}}, \tag{2}$$

with $u_{,x} = \partial u / \partial x$. Accordingly, the average state of the system is given by the functional integral

$$\langle u \rangle = \frac{1}{Z} \int_V u e^{-\beta E[u]} \mathcal{D}u. \tag{3}$$

Following the idea described in the paper, we want to approximate the function space V, over which the functional integration in (3) is carried, by a finite dimensional subspace $V^h \subset V$. To do so, we introduce a set of linearly independent functions $\{\phi_A(x)\}_{A=1}^N$ that vanish on $\partial\Omega$, such that any member of V^h is a linear combination of $\{\phi_A(x)\}_{A=1}^N$. This is the basic idea of the Galerkin method[1]. In case we want to approximate a function that does not vanish on the boundary $\partial\Omega$, but rather have some other fixed and known value there, e.g. $D(x)$ where $x \in \partial\Omega$, then we may just add this boundary condition function to the aforementioned linear combination. In case we don't want to fix the boundary, then we may add some basis functions $\phi_A(x)$ that does not vanish on the boundary (or part of it), and thus allow variation of the state there.

A FE approximation of a continuous function is a Galerkin approximation of that function, with a very specific choice of basis functions $\phi_A(x)$ that are called *shape functions*, and admit the following criteria:

1. The shape functions are related to a *mesh*. A mesh is a discretization of the spatial domain, and it comes in the form of a collection of *nodes* and *elements*. The elements are disjoint sub-domains of the geometry that together (i.e., their union) make up the geometrical domain. The nodes are points in the domain where the state value is sampled, and they interpolate the state inside each element. For example, in Supplementary Figure 1 a triangular mesh of a domain in the shape of a cat's silhouette is presented.

2. The shape function $\phi_A(x)$ vanishes at all nodes except for the A-th node, there it obtain a unit value.

3. The shape function $\phi_A(x)$ vanishes at all elements except for those containing the node A.

Soon, we will see the importance of the second and third criteria for a shape function. Before that, we classify all the nodes in the mesh to two sets. The first, is the set of nodes we call "open nodes", these are the nodes where the state value is allowed to vary, and in fact, when we take the functional integrals, we consider every possible value of the state at those nodes. This set of nodes is denoted by η. The other set of nodes are called the "closed nodes", these are the nodes where the state value is *not* allowed to vary, and throughout the functional integration these state-values at these nodes remains fixed. This set of nodes is denoted by η_u. Following these classification, and the Galerkin method, a FE approximation has the form

$$u(x) \approx u^h(x) = \sum_{A\in\eta} \phi_A(x) d_A + \sum_{A\in\eta_u} \phi_A(x) \bar{u}_A. \tag{4}$$

At first glance, equation (4) is just a simple linear combination of shape functions; however, because $\phi_A(x)$ are shape functions, then it has a very unique and important property. Suppose B is some node in the mesh, and x_B is the location of the node in Ω, the domain. Then

$$u^h(x_B) = \sum_{A\in\eta} \phi_A(x_B) d_A + \sum_{A\in\eta_u} \phi_A(x_B) \bar{u}_A = \sum_{A\in\eta} \delta_{AB} d_A + \sum_{A\in\eta_u} \delta_{AB} \bar{u}_A = \begin{cases} d_B & B\in\eta \\ \bar{u}_B & B\in\eta_u \end{cases}, \tag{5}$$

where δ_{AB} is Kroneker's delta. In words, due to the second criterion that defines a shape function, the coefficients of the linear combination in (4) are the values of the approximated function. The coefficients d_B are the variables state-values at the open nodes, and the coefficients $\bar{u}_A$ are the fixed state-values at the closed nodes.

In the paper, we chose, for simplicity, $\phi_A(x)$ to be linear shape functions. This means that $\phi_A(x)$ varies linearly from 1 to 0 inside each element containing the node A, and that it vanishes in all other elements, as depicted in Supplementary Figure 2. Therefore, the FE approximation u^h from equation (4) is actually a piece-wise-linear approximation of u between of its nodal values d_A and $\bar{u}_A$.

We continue by taking the FE approximation u^h and substituting it to the energy functional $E[u]$ from equation (2), so it becomes a function $E^h(\mathbf{d}) = E[u^h]$ of a vector variable $\mathbf{d}$. This is very straight forward, the energy integral is performed over x, but the state is now of finite-dimension, thus making E^h a regular function of a vector variable. To carry out the integral itself, it is more convenient to look at each element separately, and then take the sum of the result, i.e.,

$$E^h(\mathbf{d}) = \sum_{e=1}^{N_{el}} (E^h(\mathbf{d}))^e = \sum_{e=1}^{N_{el}} \left(\int_{\Omega^e} \frac{1}{2}\sigma(u^h_{,x})^2 \,\mathrm{d}\Omega - \int_{\Omega^e} f u^h \,\mathrm{d}\Omega \right), \tag{6}$$

where N_{el} is the number of elements in the mesh, $(E^h(\mathbf{d}))^e$ is a shorthand of the energy of element number e, and Ω^e is the subdomain of that element. We are now ready to appreciate the third criteria for a shape function; if we look back at Supplementary Figure 2, we see that there exist only two non-zero shape-functions inside each element (for example, the element between nodes A and $A+1$ in the figure). More generally, inside each element there are the same number of shape functions as there are nodes. This might seem obvious but this point that is important to stress, due to this locality property the calculation of the integral can be done over each element separately while only considering the shape functions in it.

Following this conclusion, it is very appropriate to define a vector of element shape functions,

$$\boldsymbol{\phi}(x) = \{\phi_1^e(x), \phi_2^e(x)\}^T,$$

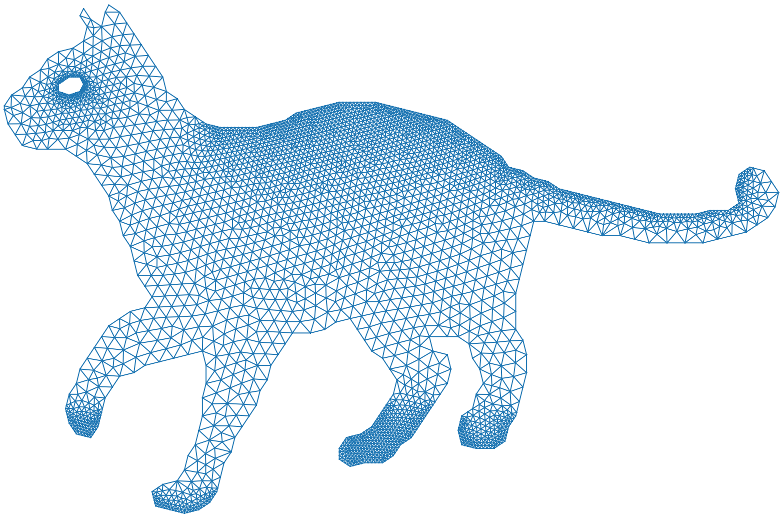

Supplementary Figure 1: Example of a non-uniform mesh over a 2-D domain in the shape of a cat's silhouette. The mesh was generated using the open-source Gmsh [7].

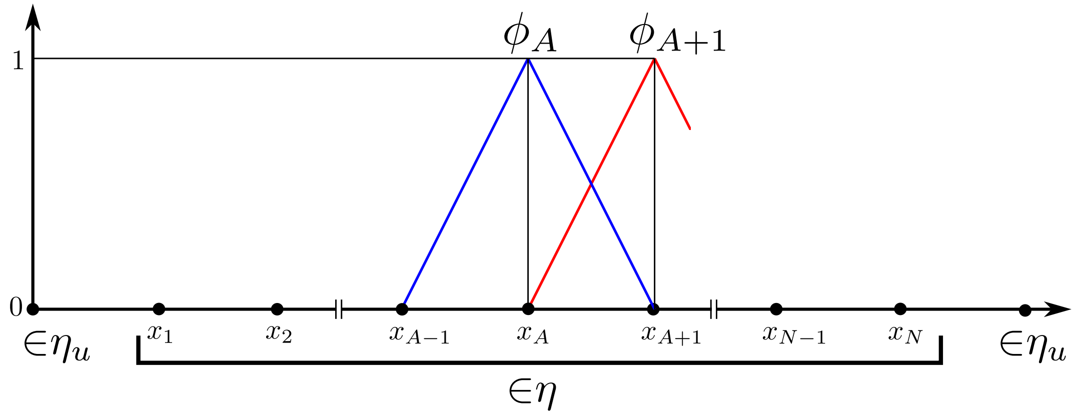

Supplementary Figure 2: Illustration of a linear shape function in a 1-D mesh.

where $\phi^e_a(x)$ are the shape functions in the element; and a vector of element state-values,

$$\mathbf{d}^e = \{d^e_1, d^e_2\}^T,$$

where d^e_a are the state-values in the element; such that state inside each element is given by $u^h = \boldsymbol{\phi}^T\mathbf{d}^e$. We note here few things:

1. We require no special treatment for elements that share nodes with the boundary. For such elements, the nodes on the element just take the fixed boundary-value, $\bar{u}_A$, into d^e_a.
2. The global state is *not* a sum of the element states, but rather it's an *assembly* of them, that would result in a dot product of all shape functions with their corresponding state- or boundary-values. The assembly process is a core concept of the finite element method and we will discuss it in greater details later in the text.

We look again at equation (6) for the subdivision of the energy integral between the elements, and use our new element-level definitions for $\boldsymbol{\phi}(x)$ and $\mathbf{d}^e$ to write the energy of an element as

$$(E^h)^e = \int_{\Omega^e} \frac{1}{2}\sigma(\boldsymbol{\phi}^T_{,x}\mathbf{d}^e)^2 \, \mathrm{d}\Omega - \int_{\Omega^e} f\boldsymbol{\phi}^T\mathbf{d}^e \, \mathrm{d}\Omega \,. \tag{7}$$

At this point, depending on the complexity of the spatial dependence of σ and f, the integrals can be either carried out exactly or by means of numerical quadrature rules over each element separately. If a numerical approach is taken for the calculation of these integral then special care should be given to ensure that the error introduced by the numerical integration is at least of the same order of the error introduced by the FE approximation. Another very useful approximation that is usually done at this point, is taking the external loading function f, which is generally a function of the spatial variable, and approximating it over the element using the FE shape functions, i.e. writing $f \approx f^h = \boldsymbol{\phi}^T\hat{\mathbf{f}}^e$, where $\hat{\mathbf{f}}^e$ is a vector whose components are the loading values at the element nodes. Next, due to our simplifying assumption regarding $f \approx f^h$, the uniform tension σ, and the linear shape functions $\boldsymbol{\phi}(x)$, we can simplify equation (7) much further,

$$(E^h)^e = \frac{1}{2}\mathbf{d}^{eT}\left(\sigma\int_{\Omega^e}\boldsymbol{\phi}_{,x}\boldsymbol{\phi}^T_{,x}\right)\mathbf{d}^e - \hat{\mathbf{f}}^{eT}\left(\int_{\Omega^e}\boldsymbol{\phi}\boldsymbol{\phi}^T\,\mathrm{d}\Omega\right)\mathbf{d}^e := \frac{1}{2}\mathbf{d}^{eT}\mathbf{k}^e\mathbf{d}^e - \hat{\mathbf{f}}^{eT}\mathbf{m}^e\mathbf{d}^e \tag{8}$$

where we defined two new quantities, the first, $\mathbf{k}^e = \sigma\int_{\Omega^e}\boldsymbol{\phi}_{,x}\boldsymbol{\phi}^T_{,x}\,\mathrm{d}\Omega$, which is called the *element stiffness matrix*, and the second, $\mathbf{m}^e = \int_{\Omega^e}\boldsymbol{\phi}\boldsymbol{\phi}^T\,\mathrm{d}\Omega$, whcih is called the *element mass matrix*. Note that we haven't applied yet the simplification regarding linear shape-functions. Therefore, the element stiffness and mass matrices provide some general structure to which we can apply any kind of element with any kind of shape functions – one only needs to calculate these element arrays to get the energy of the element. For example, for one-dimensional elements with linear shape-functions, the element arrays have the following simple formulas

$$k^e_{ab} = \frac{\sigma}{|\Omega^e|}\begin{bmatrix} 1 & -1 \\ -1 & 1 \end{bmatrix} \quad \text{and} \quad m^e_{ab} = \frac{|\Omega^e|}{6}\begin{bmatrix} 2 & 1 \\ 1 & 2 \end{bmatrix}. \tag{9}$$

Here, $|\Omega^e|$ is the length of the e-th element, which domain is Ω^e.

At this point in the analysis, if one is only interested in the numeric value of the energy (for example, to be used in a Monte Carlo simulation), then the computation should end here. However, if we want to get closer to an analytical expression, of the kind provided in equation (9) in the article, then we should *assemble* the element results into a global result. The assembly algorithm is a core concept of the finite element method, and can be found in many textbooks, notes and summaries on the subject (see for example the references mentioned in the end of this text). Simply put, the assembly process comes from the observation that the sum over all the element-energy expressions is composed of different matrices ($\mathbf{k}^e$ and $\mathbf{m}^e$) multiplying vectors with shared components ($\mathbf{d}^e$). Those vectors are also related to each other, since they all describe state-values. Thus the assembly algorithm take the sum of elements' array multiplication, and assemble them into one global matrix multiplication. A guideline for the algorithm follows

1. Initiate $\mathbf{K}$, the global stiffness matrix, the vectors $\mathbf{v}$ and $\mathbf{F}$ that are related to the contribution of prescribed displacements and of external loads, respectively, and the scalar S that corresponds solely to the contribution of boundary condition to the energy.

2. Go over all elements, their index e goes from 1 to N_{el}.

   (a) For each element, calculate k_{ab}^e and m_{ab}^e.

   (b) For each element, check the matching between the element nodes and the corresponding global nodes.

   - For every pair of open nodes in the element, a and b (where $a = b$ is allowed), add k_{ab}^e to the matching entry in the global matrix K.
   - For a pair of nodes where one is open (say a) and the other is closed (say b), add $k_{ab}^e d_b^e$ to the entry in $\mathbf{v}$ matching the global closed node. Recall that here d_b^e contains a fixed state-value from the boundary because the node b is closed.
   - For a pair of closed nodes, just add $k_{ab}^e d_a^e d_b^e$ to S.
   - For any open node b, add $\sum_{a=1}^{N_{en}} \hat{f}_a^e m_{ab}^e$ to the matching entry in $\mathbf{F}$. If the node is closed, add this to S. Here, N_{en} is the number of nodal points.

The reader may find a coded example for this algorithm in the supplementary material, specialized for the numeric example presented in the main text.[1]

Finally, after assembling all the element arrays into the global quantities, one arrive at the following quadratic expression for the energy of the system

$$E^h(\mathbf{d}) = \frac{1}{2}\mathbf{d}^T\mathbf{K}\mathbf{d} + (\mathbf{v} - \mathbf{F})^T\mathbf{d} + \frac{1}{2}S. \tag{10}$$

This quadratic energy may now be substituted into equation (1) so it becomes an off-centered Gaussian and thus $\langle \mathbf{d} \rangle$ may be calculated analytically from equation (3) in the papaer by considering the particular case of $g^h(\mathbf{d}) = \mathbf{d}$.

References

[1] T. J. R. Hughes, *The Finite Element Method: Linear Static and Dynamic Finite Element Analysis.* Dover Publications, 2000. Google-Books-ID: yarmSc7ULRsC.

[2] K. J. Bathe, *Finite Element Procedures.* Prentice Hall, 1996. Google-Books-ID: yCVOAQAACAAJ.

[3] R. E. White, *An Introduction to the Finite Element Method with Applications to Nonlinear Problems.* Wiley, Nov. 1985. Google-Books-ID: COBiQgAACAAJ.

[4] S. S. Rao, *The Finite Element Method in Engineering.* Butterworth-Heinemann, 2005. Google-Books-ID: nBgZqyepUGwC.

[5] J. S. Dokken, “The FEniCSx Tutorial.”

[6] F. Reif, *Fundamentals of Statistical and Thermal Physics.* Waveland Press, Jan. 2009. Google-Books-ID: ObsbAAAAQBAJ.

[7] C. Geuzaine and J.-F. Remacle, “Gmsh,” 2023.

[1] Note that, in this particular example, $f = 0$
